# Compression-extension transition of continental crust in a subduction zone: A parametric numerical modeling study with implications on Mesozoic-Cenozoic tectonic evolution of the Cathaysia Block

**DOI:** 10.1371/journal.pone.0171536

**Published:** 2017-02-09

**Authors:** Xuran Zuo, Lung Sang Chan, Jian-Feng Gao

**Affiliations:** 1 Department of Earth Sciences, The University of Hong Kong, Hong Kong SAR, China; 2 State Key Lab of Ore Deposit Geochemistry, Institute of Geochemistry, Chinese Academy of Sciences, Guiyang, China; Universidade de Aveiro, PORTUGAL

## Abstract

The Cathaysia Block is located in southeastern part of South China, which situates in the west Pacific subduction zone. It is thought to have undergone a compression-extension transition of the continental crust during Mesozoic-Cenozoic during the subduction of Pacific Plate beneath Eurasia-Pacific Plate, resulting in extensive magmatism, extensional basins and reactivation of fault systems. Although some mechanisms such as the trench roll-back have been generally proposed for the compression-extension transition, the timing and progress of the transition under a convergence setting remain ambiguous due to lack of suitable geological records and overprinting by later tectonic events. In this study, a numerical thermo-dynamical program was employed to evaluate how variable slab angles, thermal gradients of the lithospheres and convergence velocities would give rise to the change of crustal stress in a convergent subduction zone. Model results show that higher slab dip angle, lower convergence velocity and higher lithospheric thermal gradient facilitate the subduction process. The modeling results reveal the continental crust stress is dominated by horizontal compression during the early stage of the subduction, which could revert to a horizontal extension in the back-arc region, combing with the roll-back of the subducting slab and development of mantle upwelling. The parameters facilitating the subduction process also favor the compression-extension transition in the upper plate of the subduction zone. Such results corroborate the geology of the Cathaysia Block: the initiation of the extensional regime in the Cathaysia Block occurring was probably triggered by roll-back of the slowly subducting slab.

## 1. Introduction

Subduction is a process that dominates the dynamics of the Earth as it modifies the nature of the mantle and crust, produces arc volcanism, and releases most of the seismic energy on Earth [1]. Various processes associated with subduction such as slab development, magmatism at active margins, have been extensively studied in the past [2–8]. Regional stress configuration of continental crust would be very different in various processes or stages of subduction so that crustal stress would be changed during subduction. In most cases, the continental crust would experience the transition from compression to extension as subduction goes on, concomitant with extensive magmatism and formation of sedimentary basins [6–8]. Thus, the mechanism of such transition is critical for the understanding of the nature of paleo-subduction. Traditional methods employed in studying the active-passive margin transition, including swath bathymetry, seismic profile and stratigraphical analysis, can only be used to establish the evolution of the margin at different time stages [6, 7]. Although the direction and rate of plate movements can be roughly reconstructed by paleomagnetic data, many essential paleo-geological conditions including the geothermal gradient and the dipping angle of the slab are very difficult to determine by such traditional methods.

Numerical geodynamic modeling has been successfully applied in many studies of various scenarios of subduction [9, 10] and crustal / mantle lithosphere deformation [11, 12]. For example, the code Flamar v12 [11] can be used to stimulate how geological parameters may affect crustal stress configuration, which could be validated with geological observations. Thus, numerical modeling is a powerful tool to reconstruct the subduction system and thus the evolutionary history of crustal stress. Most of the previous studies on modeling of the switch of crustal stress from compression to extension mainly focused on the initiation of subduction at passive margins and addressed dominant controls for the transition, such as thermal buoyancy force and sedimentary loading [13–16]. Other factors controlling the process of transition from compression to extension of continental crust in the subduction zone is still poorly understood.

**Fig 1:**
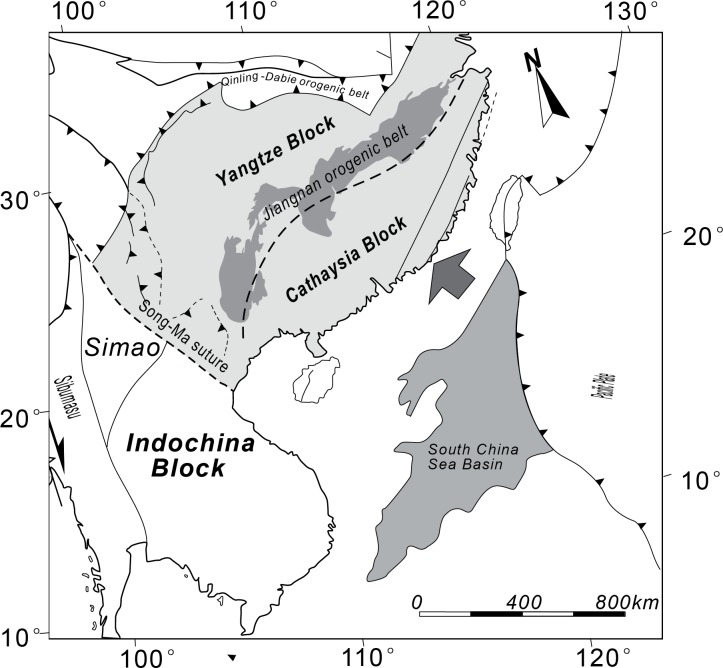
Simplified geological map of the Cathaysia Block and the surrounding areas (Modified after [26]). The NW-trending faults were reactivated during the subduction and rifting.

The Cathaysia Block is located in Southeastern China (Fig 1), which is in the upper plate of a subduction zone [17–19]. During Late Jurassic to Cretaceous, this block was subjected to the subduction of the paleo-Pacific Plate in the southeast coastal region [19–22] and the oblique subduction of the Izanagi Plate in the northeast [23], accompanied with a major regional magmatism. This block then underwent a transition from compressional to extensional setting [24], leading to the formation of the fault and basin system in the Cathaysia Block, including the reactivation of numerous NE-striking faults and the formation of oil- and natural gas-bearing basins, whose development climaxed with deposition of the Late Cretaceous-Paleocene continental red beds [18, 25–30]. In addition, long-term subduction beneath the Cathaysia Block has generated extensive granitic magmatism, forming different types of granitoids and volcanic rocks[19]. Thus, tectonic transition of the Cathaysia Block has been well recorded, making it a good example to study the mechanism and process of transition from active to passive margin by numerical modeling.

This study utilized a numerical thermo-dynamical program to address how geological parameters (e.g. variable slab angles, thermal gradients of the lithosphere and convergence velocities) may give rise to crustal deformation and thus assess the mechanism of compression-extension transition at convergent continental margins. The numerical results will be further compared with a global compilation of subduction zone parameters within the same type of subduction system. The modeling results, together with the geology of the Cathaysia Block, provide constraints on the setting of the geodynamic evolution of the subduction system during the tectonic switch of the Cathaysia Block.

## 2. Setup of numerical model

### 2.1 Code description

The thermo-mechanically and thermo-dynamically coupled numerical code Flamar v12 [11] was used to simulate the stress configuration of the subduction zone. FLAMAR is a mixed finite-element/finite difference code based on the FLAC algorithm [31]. It solves simultaneously Newtonian dynamic equations of motion, in a Lagrangian formulation. Several parameters are taken into consideration of this code: (1) large strains and visco-elasto-plastic rheologies, (2) pressure-temperature strain-rate dependent ductile creep, (3) mineralogical phase transitions, and (4) free surface boundary conditions and surface processes. The profiles of lithology, temperature, stress, strain, topography and etc. can be obtained at any moment during the model running. FLAMR has been already tested on a number of geodynamical problems for subduction/collision context [32–34].

#### 2.1.1 Basic equations

Flamar (derivative of PARAVOZ) is a FLAC-like code [31]. It has a mixed finite-difference/finite element scheme, with a Cartesian coordinate frame and a 2D plane strain formulation. The Lagrangian mesh is composed of quadrilateral elements subdivided into two couples of triangular subelements with tri-linear shape functions. Flamar utilizes a large strain fully explicit time-marching algorithm. It locally solves full Newtonian equations of motion in a continuum mechanics approximation

$$<\rho \ddot{u}>-\nabla \sigma -\rho g=0$$ (1)

coupled with constitutive equations:

$$\frac{D\sigma }{Dt}=F(\sigma ,u,\dot{u},\nabla \dot{u},\ldots T\ldots )$$ (2)

and with equations of heat transfer (heat advection term $\dot{u}\nabla T$ is included in the lagrangian derivative *DT/Dt*):

$$\frac{\rho C_{P}DT}{Dt}-k\nabla^{2}T-\sum_{i}^{n}H_{i}=0$$ (3)

ρ=f(P,T) (4)

where **u**, ***σ***, ***g***, *k* are the respective terms for displacement, stress, gravitational acceleration and thermal conductivity. P is pressure (negative for compression). The inertial term in triangular brackets in Eq (1) is negligible for geodynamic applications. It is retained since FLAC employs an artificial inertial dampening density allowing to slow-down the elastic waves and hence advance with considerably larger time steps [31] than would be required in a fully inertial mode. The terms *t*, ρ, *C*_*P*_, *T*, H_i_ designate, respectively, time, density, specific heat capacity, temperature, internal heat production per unit volume. The symbol ∑ means summation of various heat sources H_i_. The expression ρ = f(P,T) refers to the formulation in which phase changes are taken into account and density is computed by a thermodynamic module that evaluates the equilibrium density of constituent mineralogical phases for given *P* and *T* as well as latent heat contribution *H*_*l*_ to the term $\sum_{i}^{n}H_{i}\;(\sum_{i}^{n}H_{i}=H_{r}+H_{f}+H_{l}+H_{a}\ldots )$, which also accounts for radiogenic heat *H*_*r*_, frictional dissipation *H*_*f*_ and adiabatic heating *H*_*a*_. Although some studies advocate for strong efficiency of shear heating [35], in the absence of direct observational data we decided not to include the shear heating in our computation. The terms *Dσ/Dt* and *F* denote the objective Jaumann stress rate and a function, respectively. In the Lagrangian method, incremental displacements are added to the grid coordinates allowing the mesh to move and deform with the material. This allows for the solution of large-strain problems while using locally the small-strain formulation: on each time step the solution is obtained in local coordinates, which are then updated in a large-strain mode, as in a standard finite element framework.

Solution of Eq (1) provides velocities at mesh points used for computation of element strains and of heat advection $\dot{u}\nabla T$. These strains are used in Eq (2) to calculate element stresses and equivalent forces used to compute velocities for the next time step. Due to the explicit approach, there are no convergence issues, which is rather common for implicit methods in case of non-linear rheologies. The algorithm automatically checks and adopts the internal time step using 0.1–0.5 of Courant’s criterion of stability, which warrants stable solution.

#### 2.1.2 Explicit phase changes

A direct solution for density, Eq (4): *ρ* = *f*(*P*,*T*), is obtained from direct optimization of Gibbs free energy for a typical mineralogical composition of mantle and lithosphere material. The thermodynamic PERPLEX algorithm [36] has been coupled with the main code via Eq (4) to introduce progressive density changes rather than using a fixed density grid based on metamorphic facies alone. PERPLEX minimizes free Gibbs energy *G* for a given chemical composition to calculate an equilibrium mineralogical assemblage for the given P-T conditions:

$$G=\sum_{i=1}^{n}\mu_{i}N_{i}$$ (5)

where *μ*_*i*_ is the chemical potential and *N*_*i*_ the moles number for each component i constitutive of the assemblage. Given the mineralogical composition, the computation of density is straightforward [10, 37]. The thermodynamic and solid state physics solutions included in PERPLEX also yield estimations for elastic and thermal properties of the materials, which are integrated in the thermo-mechanical kernel of Flamar.

#### 2.1.3 Explicit elastic-viscous-plastic rheology

We use a serial (Maxwel-type) body for isotropic material, in which the total strain increment in each numeric element is defined by a sum of elastic, viscous and brittle strain increments. Consequently, in contrast to fluid dynamic approaches, where non-viscous rheological terms are simulated using pseudo-plastic viscous terms (e.g.[38, 39]), our method explicitly treats all rheological terms. The parameters of elastic-ductile-plastic rheology laws for crust and mantle are derived from rock mechanics data [40, 41].

a) Plastic (brittle) behavior

The brittle behavior of rocks is described by Byerlee’s law [42, 43], which corresponds to a Mohr-Coulomb material with friction angle *φ* = 30° and cohesion |*C*_0_|<20 MPa (e.g. [44])

$$|\tau |=C_{0}-\sigma_{n}tan\varphi$$ (6)

where *σ*_n_ is normal stress $\sigma_{n}=\frac{1}{3}\sigma_{Ι}+\sigma_{\Pi }^{dev}\sin\varphi ,\;\frac{1}{3}\sigma_{1}=P$ is the effective pressure (negative for compression), $\sigma_{\Pi }^{dev}$ is the second invariant of deviatoric stress, or effective shear stress. The condition of the transition to brittle deformation (function of rupture *f*) reads as: $f=\sigma_{\Pi }^{dev}+P\sin\varphi -C_{0}\cos\varphi =0$ and ∂*f*/∂*t* = 0. In terms of principal stresses, the equivalent of the yield criterion reads as

$$\sigma_{1}-\sigma_{3}=-\sin\varphi (\sigma_{1}+\sigma_{3}-2C_{0}/\tan\varphi )$$ (7)

b) Elastic behavior

The elastic behavior is described by the linear Hooke’s law

$$\sigma_{ij}=\lambda \varepsilon_{ii}\delta_{ij}+2G\varepsilon_{ij}$$ (8)

where λ and G are Lamé’s constants. Repeating indexes mean summation and δ is the Kronecker’s operator.

c) Viscous (ductile creep) behavior

Within deep lithosphere and underlying mantle regions, creeping flow is highly dependent on temperature and is non-linear non-Newtonian since the effective viscosity can vary within 10 orders of magnitude as function of differential stress [40, 43]:

$${\dot{\varepsilon }}^{d}={A(\sigma_{1}-\sigma_{3})}^{n}exp(-QR^{-1}T^{-1})$$ (9)

where ${\dot{\varepsilon }}^{d}$ is effective shear strain rate, *A* is a material constant, *n* is the power-law exponent, *Q* is the creep activation enthalpy, *R* is the universal gas constant, and *T* is the absolute temperature, σ_1_ and σ_3_ are the principal stresses. The effective viscosity *μ*_eff_ for this law is defined as

$$\mu_{eff}={\dot{\varepsilon }}^{\left(1-n\right)/n}A^{-1/n}\exp\left(Q{\left(nRT\right)}^{-1}\right)$$ (10)

For non-uniaxial deformation, the law Eq (10) is converted to a triaxial form, using the invariant of strain rate and geometrical proportionality factors

$$\mu_{eff}={\dot{\varepsilon }}_{\Pi }^{d(1-n)/n}{\left(A^{*}\right)}^{-1/n}\exp\left(Q{\left(nRT\right)}^{-1}\right)$$ (11)

where

$${\dot{\varepsilon }}_{\Pi }^{d}={\left[\ln\upsilon_{\Pi }({\dot{\varepsilon }}_{ij})\right]}^{1/2}\;and\;A^{*}=0.5A∙3^{(n+1)/2}$$ (12)

Parameters A, n, Q are experimentally determined material constants (Table 1). Using olivine parameters, one can verify that the predicted effective viscosity at the base of the lithosphere is 1019–5*10^19^ Pa∙s matching post-glacial rebound data [45]. In the depth interval of 250 km—0 km, the effective viscosity grows from 10^19^ to 10^25^−10^27^ Pa∙s with decreasing temperature. Within the adiabatic temperature interval in the convective mantle, the dislocation flow law Eq (10) is replaced by a nearly Newtonian diffusion creep, which results in a quasi-constant mantle viscosity of 10^19^−10^21^ Pa∙s (e.g. [45]).

**Table 1:** Rheological parameters used in all experiments for each unit/phase.

Natural material		Sediment	Continental crust		Oceanic crust		Mantle	
			Upper	Lower	Upper	Lower	Lithospheric	Asthenospheric
Substitutes in models		Schist	Quartz	Granulite	Olivine	Serpentine	Olivine	Olivine
Viscosity	n	31	3	4.2	3	5.8	3	3
	A (MPa^-n^·s^-1^)	1.30E-67	6.80E-06	1.40E+04	7.00E+03	2.10E-20	7.00E+03	7.00E+03
	Q (KJ·mol^-1^)	9.80E+04	1.56E+05	4.45E+05	5.10E+05	2.10E+04	5.10E+05	5.10E+05
	references	S & K [48]	R & M [49]	W & C [50]	G & E [51]		G & E [51]	G & E [51]
Elasticity	λ (Pa)	1.00E+10	3.00E+10	3.00E+10	3.00E+10	3.00E+10	4.00E+10	4.00E+10
	μ (Pa)	1.00E+10	3.00E+10	3.00E+10	3.00E+10	3.00E+10	4.00E+10	4.00E+10
Plasticity	cohesion (Pa)	1.00E+06	2.00E+07	2.00E+07	2.00E+07	1.00E+07	2.00E+07	5.00E+08
	friction angle(°)	5	30	30	10	5	30	2

#### 2.1.4 Surface erosion and sedimentation

The code handles explicit free surface boundary condition. Thus different from a number of existing codes, the surface velocity and displacement are computed in a straightforward way, without simplifying assumptions.

A simple law to simulate erosion and sedimentation is applied to the short-range surface processes associated with small-scale topography elevations (e.g. [46, 47]). Linear or nonlinear diffusion equation is expressed as:

$$\frac{\partial h_{s}}{\partial t}=\nabla (k_{e}\nabla h_{s})$$ (13)

where *h*_*s*_ and *k*_*e*_ denote surface elevation and coefficient of erosion respectively. In particular, the diffusion equation assures a number of important properties of the surface processes: (1) dependence of the local erosion rate on surface curvature and slope, so that actively deforming topography is subject to faster erosion; (2) mass conservation; and (3) smoothing of the surface with time in the absence of active subsurface deformation.

### 2.2 Initial configuration

**Fig 2:**
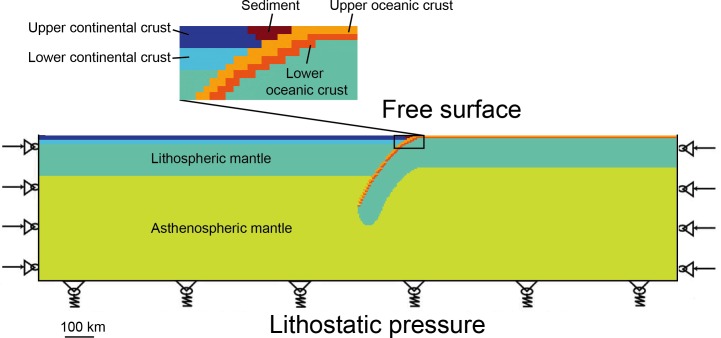
Boundary conditions applied to models (Example of the reference model).

**Fig 3:**
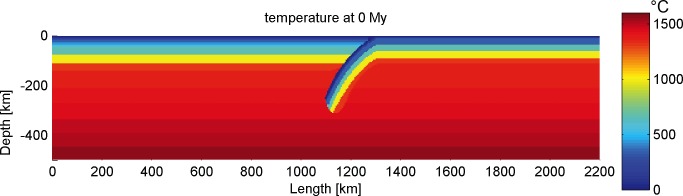
Initial thermal profile of the reference model.

**Fig 4:**
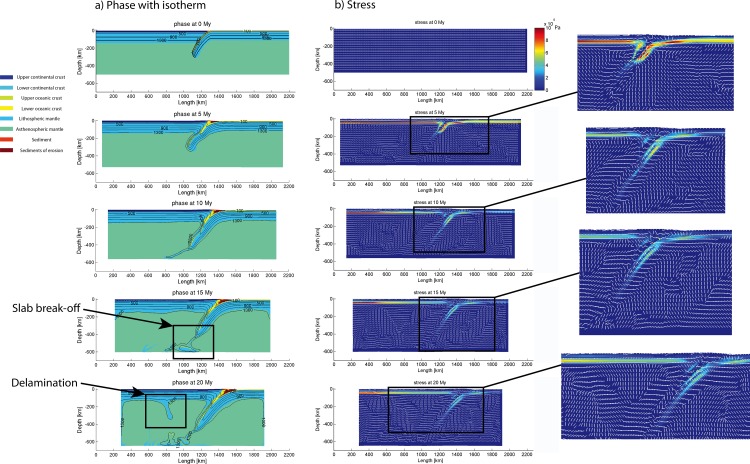
Evolution of model “subd_ref” at 0 My, 5 My, 15 My and 20 My. (a) lithological phases with isotherms in the lithosphers; (b) profiles of second invariant of deviatoric stress, indicating the effective shear stress.

In order to simulate the processes of different subduction systems, the physical parameters of the materials used were set as realistic as possible. The initial parameters include: (1) rheological and thermal parameters of materials used (Tables 1 and 2), (2) geometrical configuration (Fig 2; Tables 3 and 4) (3) geothermal gradient (Fig 3), (4) dip angle, (5) thickness of crust and mantle and (6) velocity of convergence. Among these parameters, rheological and thermal parameters of materials are set as constant values in the program (Tables 1 and 2). The thicknesses of different crustal units (Table 3) for the reference model (subd_ref) are derived from the modern Cathaysia Block. As we don’t know the status of Cathaysia Block, we also designed some reasonable variations of the crustal thickness (Table 4) to explore other possibilities.

**Table 2:** Thermal parameters and densities of adopted materials.

**Thermal parameters**	
Surface temperature (0 km depth), *T*_*0*_	0°C
Temperature at the bottom of lithosphere, *T*_*m*_	1330°C
Temperature at 500km depth	1450°C
Thermal conductivity of crust, *k*_*c*_	2.5 W m^-1^°C^-1^
Thermal conductivity of mantle, *k*_*m*_	3.3 W m^-1^°C^-1^
Specific heat capacity	1*10^3^J kg^-1^°C^-1^
Surface radiogenic heat production, *H*_*s*_	5*10^−9^ W kg^-1^
Radiogenic heat production decay depth, *h*_*r*_	12 km
Thermotectonic age of oceanic/continental lithosphere, *a*	250 / 500 Ma
**Material densities**	
Continental upper crust	2750 kg m^-3^
Continental lower crust	2980 kg m^-3^
Oceanic crust	2900 kg m^-3^
Sediment	2600 kg m^-3^
Lithospheric mantle	3300 kg m^-3^
Asthenospheric mantle	3330 kg m^-3^

**Table 3:** Material thickness in the reference model.

Natural material	Thickness (km)
Upper continental crust	15
Lower continental crust	15
Continental lithospheric mantle	110
Upper oceanic crust	5
Lower oceanic crust (serpentine)	5
Oceanic lithospheric mantle	100

**Table 4:** Parameter values used for numerical experiments.

Parameters	Values tested				
Slab dip angle (°)	30	**50**		60	
Continental thermal gradient (°/km)	**9.5**	16.6		22.2	
Continental lithosphere thickness (km)	**140**	80		60	
Oceanic thermal gradient (°/km)	**12.1**	26.6		33.3	
Oceanic lithosphere thickness (km)	**110**	50		40	
Velocity of convergence (cm/yr)	2	**3**	4.5	6	12

Note: Bold numbers are the values used for the reference model (subd_ref, Table 5). Because the temperature at the lithosphere-asthenosphere boundary is set as 1330°C, the thickness of lithosphere in each model can be calculated according to the thermal gradient.

The initial geotherm of the model is vertically divided into 2 parts (Fig 3): (1) The temperature varies in a non-linear gradient from 0º on the surface to 1330ºC (1600 K) at the lithosphere/asthenosphere boundary as the conventional value [45]; (2) The temperature of mantle increases linearly with depth until 1440ºC at the depth of 440 km. In the models with different lithospheric geothermal gradients, the initial temperature gradient in the asthenospheric mantle varies from 1 to 3°C/km. There is no horizontal thermal flux from the two sides of the model box.

The dimension of the model is 2200 km×500 km and the initial geometry (Fig 2) corresponds to an ongoing subduction process. The spatial resolution (meshing) of the models is 5×5 km.

### 2.3 Boundary conditions

In the numerical experiments conducted, the boundary conditions applied are as demonstrated in Fig 2:

The surface of the models is kept as a free boundary and is modified only by erosion and sedimentation. We considered values for erosion coefficient of 1000 m^2^/year that yield denudation rates of the order of those predicted by previous parametric models (e.g. [52]) for convergence rates characterizing Eurasia-Pacific subduction.

On the side boundaries, constant speeds are applied to the nodes of mesh in the horizontal direction. For convergent settings, it does not make a difference whether the total shortening velocity is applied to one side or to both sides of the models [53]. Hence, in this model, the rates considered equivalent to the convergence velocity are applied to both sides of the model, which means that each side shares half of the effective full convergence velocity. No speed is imposed on any nodal points inside of the model as the modeling conducted by [54] and [55]. No basal shear is applied to the subduction zone in the lithosphere so that it is free to grow.

At the bottom of the model, we apply Winkler's pliable basement (i.e., hydrostatic equilibrium) with free horizontal slip condition. The Winkler's condition is such that the model overlies an infinite space filled with an inviscible fluid having a small density contrast (10 kg m^-3^) with the lower part of the model (Fig 2). The boundaries of vertical velocities are left free, thus the shortening related to laterally applied velocity would result in downward movement of the base.

### 2.4 Parameters tested

The configuration of the reference model is set up using the representative parameter values (Table 4) based on the parameters for subduction zones in the peri-Pacific region as reported by [56] and [57]. By varying initial geometry, thermal property of the lithosphere and kinetic parameters, we examined the effects of different parameters on the reference model. All parameter values used in this study for every model are presented in Tables 4 and 5 respectively. The ranges of the parameter values were chosen according to published databases [56, 57].

**Table 5:** Values of geometrical, thermal, physical and kinematic parameters of subduction models used for investigating the general development of subduction zone.

	Initial slab dip angle (°)	Thermal gradient (°C/km)		Convergence velocity (cm/yr)
		Continent	Ocean	
subd_ref	50	9.5	12.1	3
subd_v1	50	9.5	12.1	2
subd_v2	50	9.5	12.1	6
subd_v3	50	9.5	12.1	12
subd_v4	50	9.5	12.1	4.5
subd_therm1	50	16.625	26.6	3
subd_therm2	50	22.2	33.3	3
subd_angle1	30	9.5	12.1	3
subd_angle2	60	9.5	12.1	3
subd_angle1_therm1	30	16.625	26.6	3
subd_angle1_therm2	30	22.2	33.3	3
subd_angle2_therm1	60	16.625	26.6	3
subd_angle2_therm2	60	22.2	33.3	3
subd_angle1_v1	30	9.5	12.1	2
subd_angle1_v2	30	9.5	12.1	6
subd_angle1_v3	30	9.5	12.1	12
subd_angle1_v4	30	9.5	12.1	4.5
subd_angle2_v1	60	9.5	12.1	2
subd_angle2_v2	60	9.5	12.1	6
subd_angle2_v3	60	9.5	12.1	12
subd_angle2_v4	60	9.5	12.1	4.5
subd_therm1_v1	50	16.625	26.6	2
subd_therm1_v2	50	16.625	26.6	6
subd_therm1_v3	50	16.625	26.6	12
subd_therm1_v4	50	16.625	26.6	4.5
subd_therm2_v1	50	22.2	33.3	2
subd_therm2_v2	50	22.2	33.3	6
subd_therm2_v3	50	22.2	33.3	12
subd_therm2_v4	50	22.2	33.3	4.5
subd_angle1_therm1_v1	30	16.625	26.6	2
subd_angle1_therm1_v2	30	16.625	26.6	6
subd_angle1_therm1_v3	30	16.625	26.6	12
subd_angle1_therm1_v4	30	16.625	26.6	4.5
subd_angle1_therm2_v1	30	22.2	33.3	2
subd_angle1_therm2_v2	30	22.2	33.3	6
subd_angle1_therm2_v3	30	22.2	33.3	12
subd_angle1_therm2_v4	30	22.2	33.3	4.5
subd_angle2_therm1_v1	60	16.625	26.6	2
subd_angle2_therm1_v2	60	16.625	26.6	6
subd_angle2_therm1_v3	60	16.625	26.6	12
subd_angle2_therm1_v4	60	16.625	26.6	4.5
subd_angle2_therm2_v1	60	22.2	33.3	2
subd_angle2_therm2_v2	60	22.2	33.3	6
subd_angle2_therm2_v3	60	22.2	33.3	12
subd_angle2_therm2_v4	60	22.2	33.3	4.5

## 3. Results

### 3.1 Reference model

Some geometrical and kinematic parameter values have been selected for the reference model in this study, i.e., slab dip angle at 50°, continental lithosphere thermal gradient at 9.5°/km, oceanic lithosphere thermal gradient at 12.1°/km, convergence velocity at 3 cm/year (Table 4). The evolution of the reference model “subd_ref” during the numerical simulation is shown in Fig 4. Under a constant convergence velocity at 3 cm/year, this model experienced slab break-off and delamination of the lower continental lithosphere (Fig 4A). The oceanic lithosphere subducted into the mantle and preserved its original slab dip angle through the entire simulation (Fig 4A). When there is mantle upwelling under the continental lithosphere (Fig 4A), the stress regime in the continental crust is generally extensional (Fig 4B).

In general, under constant convergent velocity and slab angle, the model bears constant crustal compression in the overriding plate. Parameter values in the reference model are selectively changed in determining how the subduction parameters may modify the crustal stress configuration.

### 3.2 Convergence velocity

**Fig 5:**
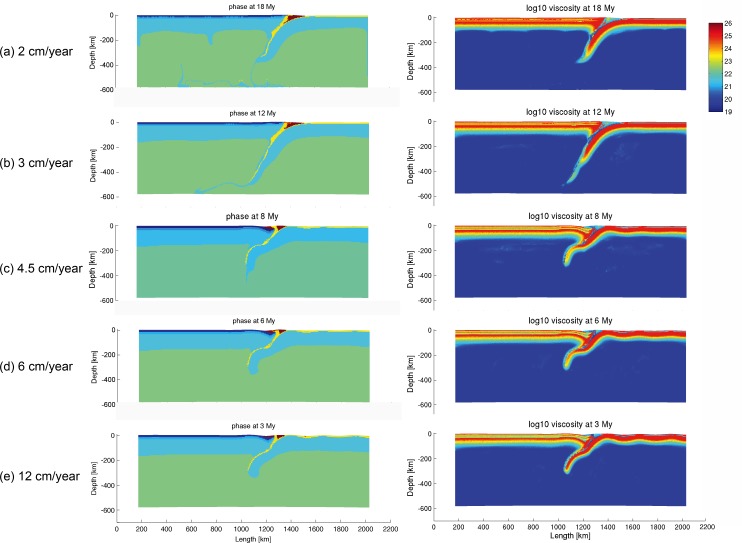
Comparison of models at different velocities with the same amount of shortening (360 km). Models with velocity at (a) 2 cm/year; (b) 3 cm/year, (c) 4.5 cm/year, (d) 6 cm/year and (e) 12 cm/year correspond to “subd_v1”, “subd_ref”, “subd_v4”, “subd_v2” and “subd_v3” respectively in Table 5.

Convergence velocities ranging from 2 cm/year to 12 cm/year were employed in the numerical modeling. The evolutions of models with the same amount of shortening (360 km) were selected for comparison (Fig 5) at convergence rate of 2 cm/year, 3 cm/year, 4.5 cm/year, 6 cm/year and 12 cm/year respectively. As shown in Fig 5A and 5B, models with low velocities underwent smooth subduction processes. However, the model with higher convergence velocities at 4.5 cm/year, 6 cm/year and 12 cm/year (Fig 5C–5E) shows buckling in the oceanic plate at 8 My, 6 My and 3Ma respectively, which indicates strong rheological coupling (defined as the effective strength of rocks composing the plate interface) at the subduction zone. As shown by the viscosity profiles, the faster the subduction process is, the more coupling at the subduction zone. The result of numerical models suggests that, high convergence velocity probably yields a strong rheological coupling, thus retarding the subduction process [58]. Slow rate of convergence, on the other hand, favors a swift subduction process. This is consistent with previous seismological studies [59, 60] showing that coupling can be enhanced by fast subduction rate.

### 3.3 Geothermal gradient of continental/oceanic lithosphere

**Fig 6:**
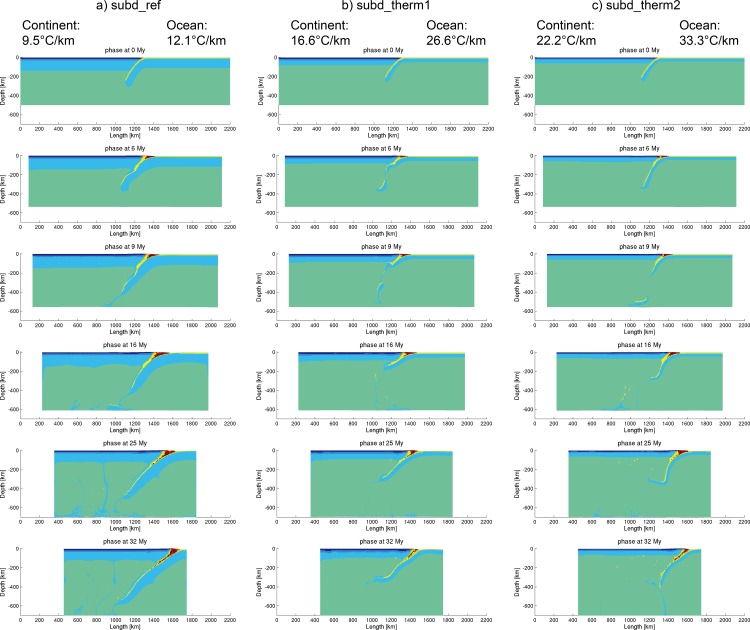
Comparison of models with different thermal gradients.

Since the conventional value of temperature at the lithosphere/asthenosphere boundary is 1330ºC [45], the thickness of lithosphere varies with the thermal gradient (i.e., the higher the geothermal gradient is, the thinner the lithospheric thickness is) (Fig 6). Based on the setting of the reference model, two more datasets of thermal gradients of continental and oceanic lithospheres were tested (Table 4), using the reference setting of initial slab dip angle (50º) and convergence velocity (3 cm/year).

The model “subd_therm1” (geotherm: 16.6°C/km for continent and 26.6°C/km for ocean) displayed a slab break-off at 6 My, developed as a low-angle subduction afterward and started flat-slab subduction at 25 My (Fig 6B). Comparably, the model “subd_therm2” (geotherm: 22.2°C/km for continent and 33.3°C/km for ocean) had a slab break-off at 9 My, evolved into a high-angle subduction, started flat-slab subduction at 16 My and then had a second slab break-off at 32 My, tending to roll-back (Fig 6C). It is obvious that the “slab break-off” easily occurs in models with higher geothermal gradients, because the thinner lithospheres break up more easily than the thicker ones (e.g. Fig 6B compared with Fig 6A). “Flat-subduction” sometimes occurs after the slab break-off, probably caused by the unloading of slab pull. However, further tests of these three sets of geothermal gradients show that high geothermal gradients do not favor a readily going subduction process when other parameters are changed, such as the convergence velocity and the initial slab dip angle. The plausible results will be summarized in the discussion.

### 3.4 Initial slab dipping angle

According to the global subduction zone database [57], the slab dip angle mainly varies from 32° to 58°. To evaluate the role of the initial slab dip angle in subduction zone, three models with initial slab dipping angles at 30°, 50° and 60° were tested, using the reference setting of thermal gradients of oceanic (12.1°C/km) and continental (9.5°C/km) lithospheres and convergence velocity (3 cm/year).

**Fig 7:**
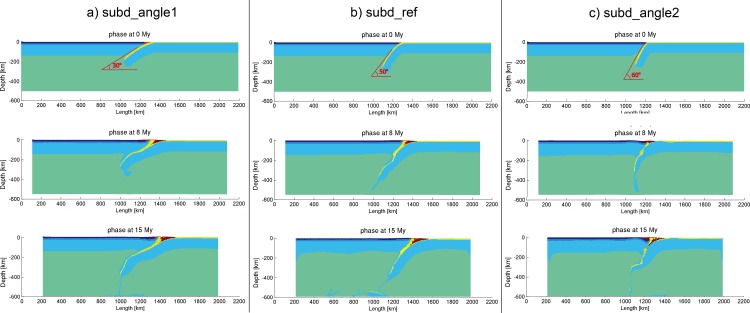
Model evolution of the models “subd_angle1”, “subd_ref” and “subd_angle2”.

As shown in Fig 7A, the model with the smallest slab dip angle got severe coupling in the subduction zone at a very early stage. Meanwhile, the bigger slab angle subduction models (Fig 7B and 7C) sustain more fluent process than the low slab angle subduction (Fig 7A). For the low angle subduction model (Fig 7A), large contact area between the subducting plate and the upper plate might be a main reason to yield more friction and to make the subducting slab more difficult to proceed forward. The model with the greatest slab dip angle (Fig 7C) shows a decreasing slab angle after the slab break-off. Thus, high angle subduction models are prone to undergo slab roll-back and break-off, due to greater influence of the mantle convection on the subducting slab.

The parameter values adopted in this series of models for convergence velocity and geothermal gradients are the same as those used the reference model. In the following sections, models for different initial slab dip angles are conducted and further elaborated with other settings of lithospheric thermal gradient and convergence velocity.

### 3.5 Combined effects of multiple parameters

The previously presented models on three types of parameters were obtained by changing only one parameter of the reference model. In order to study in greater detail the subduction mechanism, the evolution of the reference model was also tested by varying simultaneously pairs of parameters.

#### 3.5.1 Slab dip angle–Geothermal gradient

**Fig 8:**
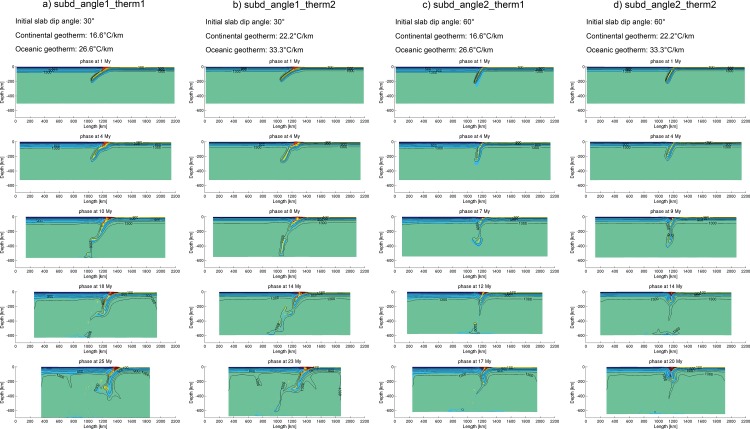
Model evolution of “subd_angle1_therm1”, “subd_angle1_therm2”, “subd_angle2_therm1” and “subd_angle2_therm2”.

The tests in this section adopted a moderate convergence velocity as in the reference model: 3 cm/year, because this is the most commonly observed convergence velocity in the peri-Pacific subduction zones [57]. Models (Fig 8A–8D) with different slab dip angles (30° and 60°) and variable geothermal gradients (16.6°C/km and 22.2°C/km for continental lithosphere; 26.6°C/km and 33.3°C/km for oceanic lithosphere) exhibit the following features:

1. The slab dip angles increase during the simulation and probably retreat, which are sometimes coeval to the mantle upwelling;
2. The slabs with higher geothermal gradients (thinner lithosphere) are easily broken-off. The thinner lithosphere might be less resistant to the mantle flow;
3. The mantle flow acting on the slab with greater dip angle can push the subducting slab to retreat. As a result, when a thin slab (i.e. very high geothermal gradient) subduct in high dip angle, the double-side subduction is prone to develop after the slab roll-back (Fig 8D).

#### 3.5.2 Slab dip angle–Convergence velocity

**Fig 9:**
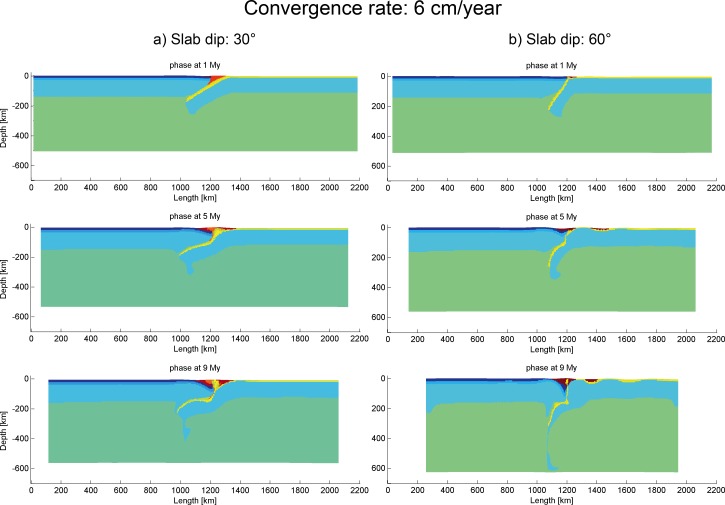
Evolution of high convergence velocity models with different slab dip angles. (a) 30° (“subd_angle1_v2”) and (b) 60° (“subd_angle2_v2”).

Models described in this section are based on low thermal gradients of the lithospheres (9.5°C/km for continent and 12.1°C/km for ocean). The conclusion of Section 3.2 and 3.4 is that higher slab dip angle and lower convergence velocity normally facilitate the progress of subduction. The models in Section 3.2 with convergence velocity higher than 6 cm/year do not work and end up with strong coupling of the subduction zone. In this section, studies on slabs with different initial dip angles (30° and 60°) at high convergence velocity (6 cm/year) still could not produce any efficient subduction process (Fig 9).

**Fig 10:**
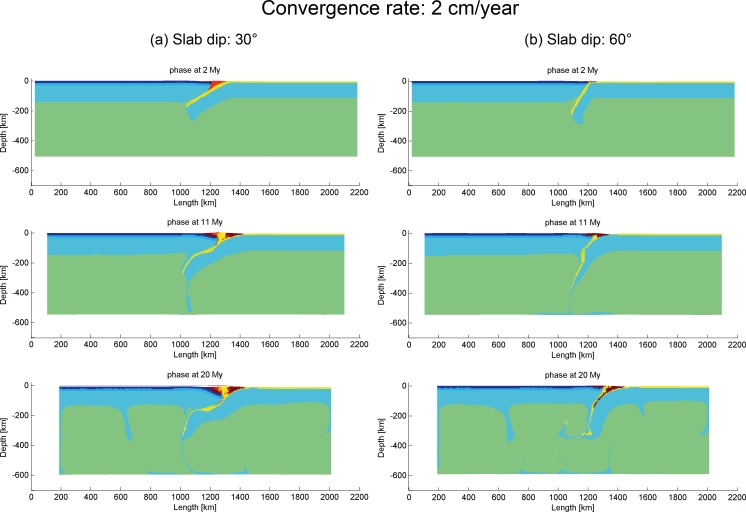
Evolution of low convergence velocity models with different slab dip angles. (a) 30° (“subd_angle1_v1”) and (b) 60° (“subd_angle2_v1”).

Distinguished from the high velocity models, subduction process can take place readily at low convergence velocity with different slab dip angles (Fig 10). The model with low slab angle (“subd_angle1_v1”) encountered some difficulty in subducting since around 20 My, while the model with high dip angle (“subd_angle2_v1”) maintained a stable subduction with the slab dip angle increasing in value with time (Fig 10).

Based on the results of the models in this section, high convergence velocity (equal to or faster than 6 cm/year) does not produce effective subduction at low geothermal gradients. Models with higher geothermal gradients will be further explored.

#### 3.5.3 Geothermal gradient—Convergence velocity

Models were designed to investigate the role of different geothermal gradients and convergent velocities at the same time. They are based on the slab dip angle of 50° as employed in the reference model.

**Fig 11:**
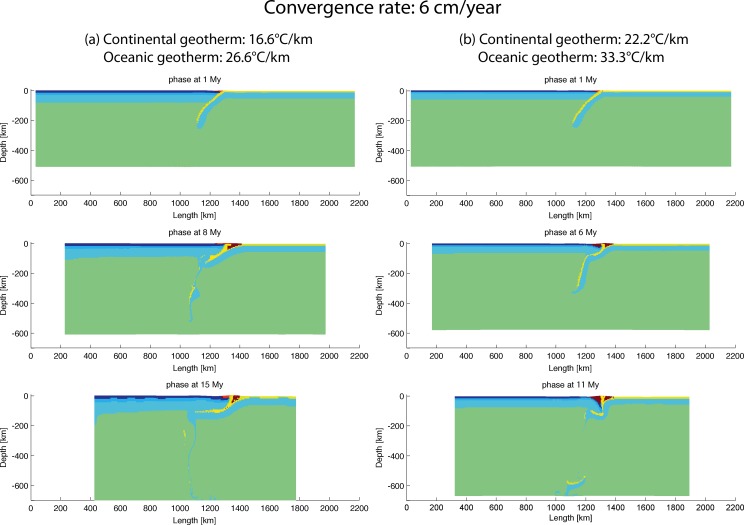
Evolution of high convergence velocity (6 cm/year) models with different geothermal gradients. (a) 16.6°C/km for continent and 26.6°C/km for ocean (“subd_therm1_v2”) and (b) 22.2°C/km for continent and 33.3°C/km for ocean (“subd_therm2_v2”). There is slab break-off at 8 My for the model “subd_therm1_v2” and at 11 My for the model “subd_therm2_v2”.

To examine the high convergence velocity (6 cm/year) models with varying geothermal gradients, two models “subd_therm1_v2” (geotherm: 16.6°C/km for continent and 26.6°C/km for ocean) and “subd_therm2_v2” (geotherm: 22.2°C/km for continent and 33.3°C/km for ocean) were created. As shown in Fig 11, both models display a slab break-off during subduction. The model “subd_therm1_v2” with medium geothermal gradients continued with a flat-slab subduction and probably mantle-derived magma underplating underneath the continental crust (phase at 15 My in Fig 11A). Dissimilarly, in the model “subd_therm2_v2” with high geothermal gradients, the subduction ceased, probably because the slab was too thin to be resistant to the compressional force coming from the continental lithosphere (phase at 11 My in Fig 11B). Therefore, the geothermal gradient contrast between oceanic plate and continental plate (i.e. the contrast of lithosphere thickness between ocean and continent) is crucial to the evolution of the subduction. For a medium slab dip angle, if the geothermal gradient of the oceanic plate is very high, it might produce a strong coupling of the subduction zone as shown in model “subd_therm2_v2” (Fig 11B).

**Fig 12:**
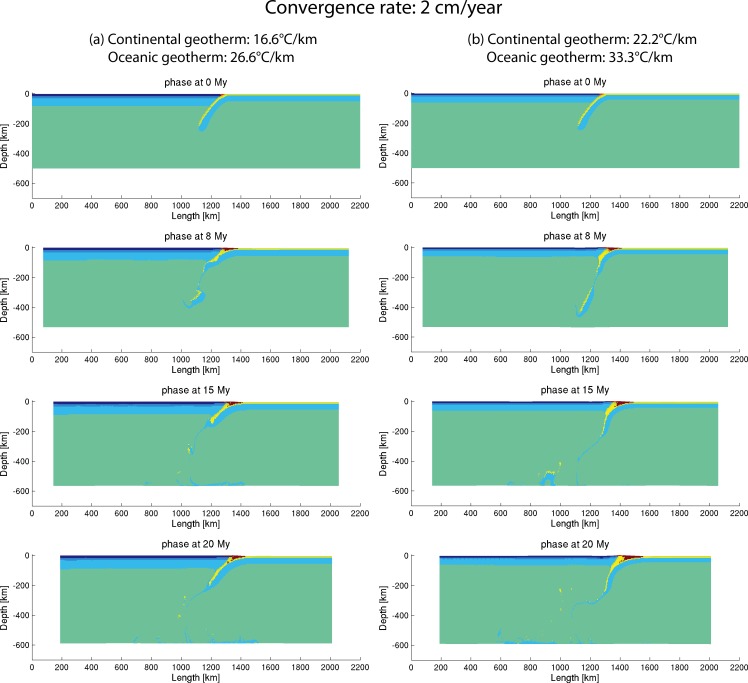
Evolution of low convergence velocity (2 cm/year) models with different geothermal gradients. (a) 16.6°C/km for continent and 26.6°C/km for ocean (“subd_therm1_v1”) and (b) 22.2°C/km for continent and 33.3°C/km for ocean (“subd_therm2_v1”).

The above-mentioned geothermal gradients were also tested based on the model with a low convergence velocity of 2 cm/year (Fig 12). Both model results show the process of slab break-off at early stage and then fluent subduction at late stage (Fig 12), while the slab in the model with higher geothermal gradients (“subd_therm2_v1”) steepened and showed more potentials of fluent evolution (Fig 12B).

Since medium geothermal gradients seem to be a favorable parameter for continent-ocean subduction models, two more values of convergence velocity for high geothermal gradients (22.2°C/km for continent and 33.3°C/km for ocean) are tested: one is a extremely fast, 12 cm/year, named as “subd_therm2_v3”; the other one is moderate, 4.5 cm/year, named as “subd_therm2_v4”.

**Fig 13:**
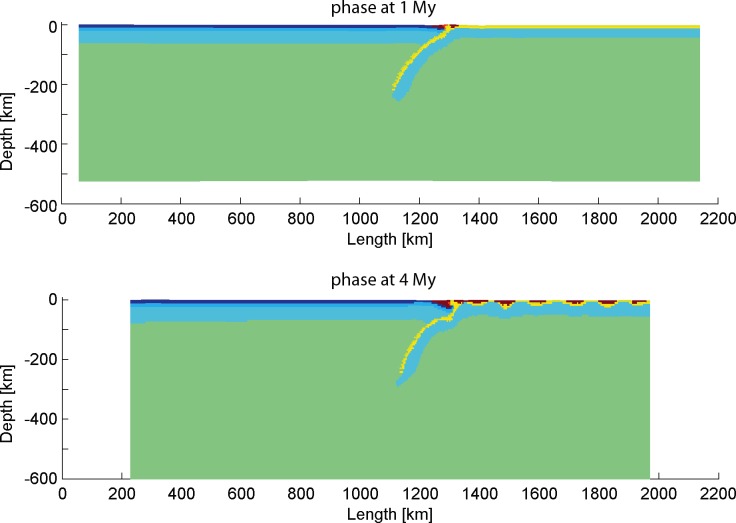
Model evolution of “subd_therm2_v3”.

**Fig 14:**
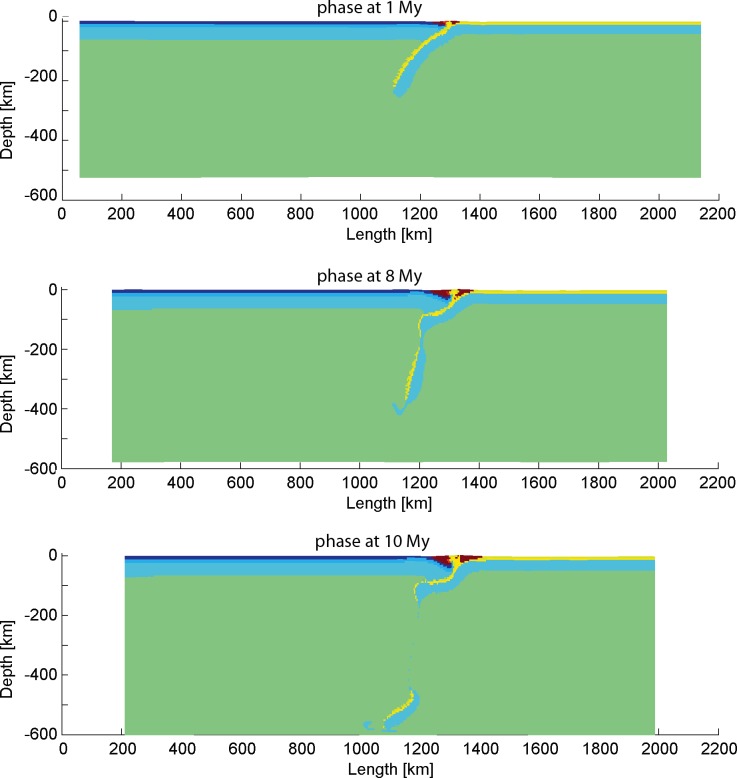
Model evolution of “subd_therm2_v4”.

As shown in Fig 13 (“subd_therm2_v3”), the subduction process is severely blocked at an early stage. The model “subd_therm2_v4” produced a slab break-off once and then the subduction stopped owing to strong coupling at the subduction zone (Fig 14), indicating that fast subduction is not suitable for subduction under high geothermal gradient conditions and a critical value of convergence rate for high geothermal gradient models should be between 3 cm/year and 4.5 cm/year.

The systematic modeling results of subduction zone in this study show that the slab dip angle is a key factor controlling the roll-back of the subducting slab. A relatively high convergence velocity is shown to produce a strong coupling of the subducting slab with the overriding plate, and a higher mantle thermal gradient would facilitate the rolling back of the slab and sometimes the flattening of slab.

## 4. Discussion

Different parameters in the numerical modeling of subduction zone, such as thermal gradient of lithosphere, slab angle, density of lithosphere and velocity of convergence, have shown variable spatial and temporal patterns of the continental deformation. Comparable with the geological observations of current subduction zones [56, 57], the numerical model results could be used for explaining the role of parameters such as slab angle, convergence velocity and geothermal gradients (thickness of continent) in the paleo-geological processes.

### 4.1 Feasible parameters of the subduction model

**Fig 15:**
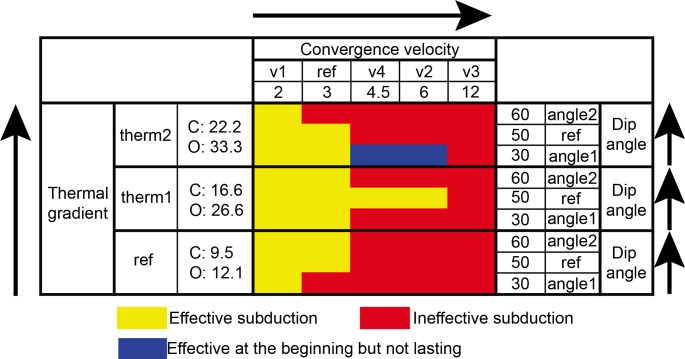
Summary of conducted subduction models.

Feasible parameters for the subduction model can be deduced from the systematic modeling of all parameter combinations (Fig 15). Different parameters play different roles in the subduction process.

#### 4.1.1 Roles of subduction slab angle

Higher slab dip angle allows the mantle to convect in a larger space. Slabs with high dip angle can roll back easily and then trigger large-scale mantle upwelling, which eventually lead to the initiation of back-arc extension. Meanwhile, the slab dip angles are in turn controlled by the mantle flow through lateral pressure on the slab [61]. Previous studies propose that the slab angle increases with time before the slab reaches the 670-km discontinuity [2]. We can infer that if the subduction zone is not coupled at the beginning of the convergence, the slab dip angle will increase, favor the steady subduction process, and eventually lead to extension in the upper plate.

#### 4.1.2 Roles of convergence velocity

For a high thermal gradient model, low slab dip angle favors the subduction and vice versa. The relatively high convergence velocities are shown to produce a strong coupling of the subducting slab with the overriding plate. The slabs in the models with very low convergence velocities have much more time to sink down and roll-back, which will sometimes result in the subduction of continental plate under oceanic plate (i.e. obduction).

#### 4.1.3 Roles of thermal gradient

For efficient subduction processes, the thermal gradients could not be too high or too low. Subduction zones with low thermal gradients (thick lithosphere) need more mantle upwelling to produce slab roll-back. Oceanic slabs with high thermal gradient (thin lithosphere) get break-off easily, because they are less resistant to the force of mantle convection.

In summary, the modeling results from the present study show that higher initial dipping angle of the subducting slab (50–60°), lower convergence velocity (2–4.5 cm/year) and medium thermal gradient of the oceanic lithosphere (about 10–20°C/km) are favorable to the subduction process.

### 4.2 Roles of slab dip angle and convergence velocity in changing continental crust stress field

**Fig 16:**
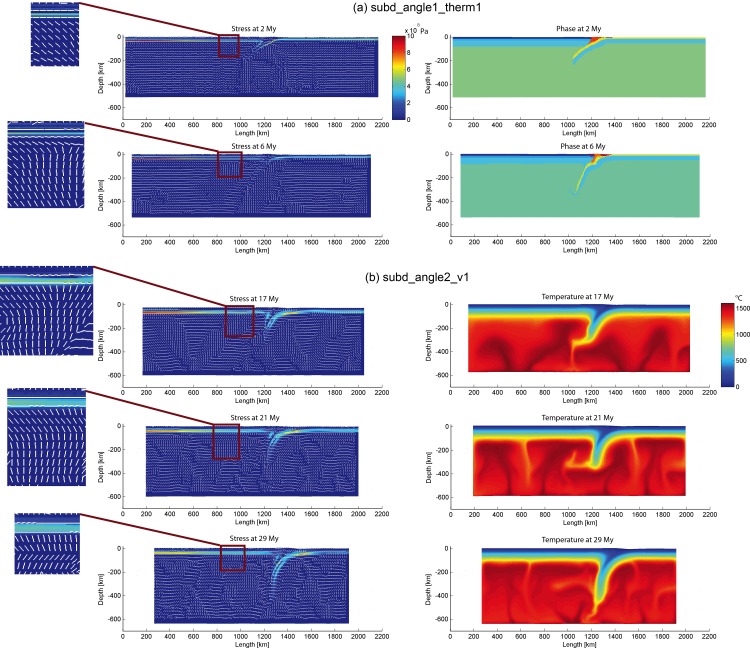
**Model evolution of “subd_angle1_therm1” and “subd_angle2_v1”:** (a) Stress field and phase lithological phase of model “subd_angle1_therm1” at 2 My and 6 My; (b) stress field and temperature state of model “subd_angle2_v1” at 17 My, 21 My and 29 My. The white bars in the stress field profiles at the left side represent the direction of the principle deviatoric stresses (σ1). Therefore, the outlined areas are mainly controlled by extensional regime.

As shown by the stress field profiles of some models, continental crust stress configuration varies significantly with different slab dip angles and convergence velocity (Fig 16). Continental crustal extension occurred to models with increasing slab dip angle. On the contrary, a low-angle subduction generally facilitates the transmission of compressive stress to the overriding plate through increased contact area between the plates. The results are consistent with some previous modeling results [60,62,63] and corroborate the inferences on the control of the strain regime of the upper plate by the slab dip angle reported previously [60,64–66]. Through a compilation of all the actual oceanic subduction zone data of upper plate absolute motion, trench absolute motion, back-arc deformation rate, upper plate strain regime and slab age, Heuret and Lallemand [56,57] examined how combined effects of these parameters can account for the observed back-arc deformations. This statistical work of current subduction zone [57,67], in particular, presents a means for testing the effects of slab angles on strain regime. The database, however, contains about 1/3 of transects located close to triple junctions where the strain configuration could have been modified by kinematic motions along other plate boundaries. The global database of subduction zones are therefore reduced by including only and transects of continent-ocean subduction zones located away from triple junctions for our analysis (Table 6).

**Table 6:** Subduction transects data used in this study (Extracted from [57]).

Name	Slab dip angle		Convergent velocity		Upper plate strain
	Shallow, °	Deep, °	Vc, mm/a	Vcmp, mm/a	
Andaman (ANDA)	34	70	18	3	E3
	33	70	18	5	0
	33	56	20	15	0
Sumatra (SUM)	29	40	41	41	0
	28	40	28	28	0
	28	49	37	37	0
	28	49	38	38	0
	28	61	39	39	0
	27	63	50	50	0
Java (JAVA)	27	71	53	53	0
	26	68	57	57	0
	28	68	60	60	0
	29	69	62	62	0
	30	68	64	64	0
	29	68	65	65	0
	27	70	66	66	0
Philippines (PHIL)	35	-	37	95	C1
	36	-	36	80	0
	34	-	42	71	0
Ryukyu (RYU)	34	58	87	55	E2
	35	61	82	53	E2
	39	64	73	48	E2
Nankai (NAN)	12	-	48	48	C1
	15	-	42	42	C1
	19	-	37	37	C1
Japan (JAP)	26	31	93	101	C3
	24	30	92	101	C3
	23	29	86	96	C3
	19	25	90	99	C3
South Kuril (SKOUR)	24	32	77	77	C2
	27	33	74	74	C2
	30	40	76	76	C2
	31	44	71	71	C2
	33	47	77	77	C2
North Kuril (NKOUR)	35	48	79	79	C1
	36	50	77	77	C1
	37	51	77	77	C1
Central Aleutian (C_ALE)	40	56	32	32	C1
	40	57	42	42	C1
	39	57.5	51	51	0
	38	58	61	61	0
	36	59	61	61	0
	35,5	62	59	59	0
East Aleutian (E_ALE)	35	61	64	64	0
	34	60	65	65	0
	33	59	65	65	E1
	32	-	64	64	E1
	31	53	33	33	0
West Alaska (W_ALA)	29	-	62	62	0
	28	48	60	60	0
	26	-	59	59	0
	24	-	58	58	0
	23	45	58	58	0
East Alaska (E_ALA)	21	43	56	56	C1
	19	40	52	52	C1
	18	38	47	47	C1
Cascadia(CASC)	13	45	31	32	0
Mexico (MEX)	20	-	51	51	E1
	16	-	55	55	E1
	18	-	60	60	E1
	25	-	61	61	E1
Costa-Rica (COST)	27	55	63	63	E1
	28	54	68	68	0
	30	64	73	73	0
	32	66	78	78	0
Colombia (COL)	21	45	42	55	C3
Peru (PER)	11	45	69	69	C3
	10	46	71	71	C3
	10	47	71	71	C3
	11	49	70	70	C3
	12	49	68	68	C3
	12	52	68	68	C3
North Chile (NCHI)	17	50	58	58	C3
	25	41	63	73	C3
	23	40	72	78	C3
	18	45	71	75	C3
	17	47	71	77	C3
	14	49	68	73	C3
Juan Fernandez (JUAN)	13,5	-	69	75	C3
	12	-	72	77	C3
South Chile (SCHI)	21	35	66	66	C1
	22	-	62	62	C1
	24	-	72	72	C1
	25	-	75	75	C1
	25	-	75	75	C1
Antilles (ANT)	39	-	11	11	E1
	37	-	9	9	E1
	35	-	6	6	E1
Proto-Antilles (PORTO)	32	-	8	3	E1
	35	-	5	1	E1

Note: Vc is the effective convergence at trench, Vcmp is the convergence between major plates. All the velocities are normal component of the absolute velocities, corresponding to the velocities in 2D models. The upper plate strain is characterized by significant active extension (E3) to significant active compression (C3).

**Fig 17:**
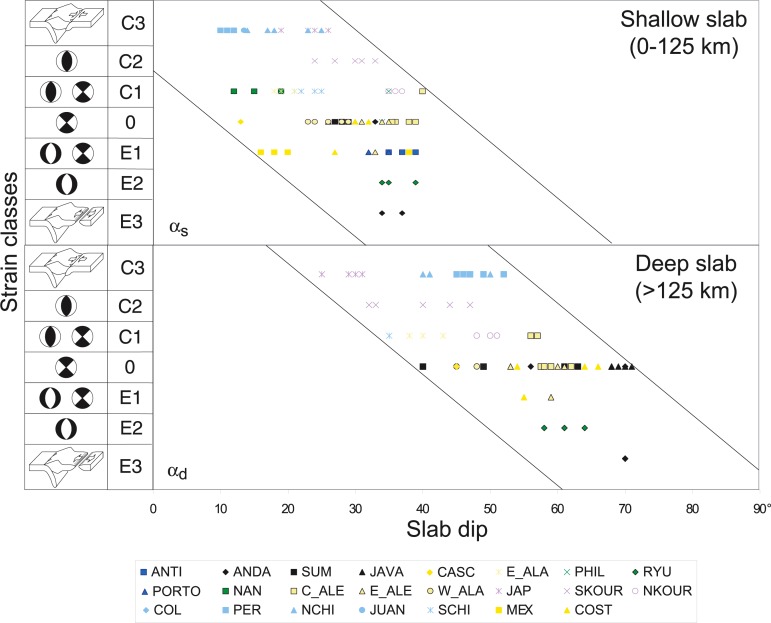
Correlation between slab dip and upper plate strain (Modified after [57]). Abbreviations for subduction zones names are given in Table 6.

According to the approach of Jarrard [65], the transects are classified by strain characteristics within the upper plate from significant active extension (class E3) to significant active compression (class C3). The correlation between slab dip and upper plate strain is plotted for the reduced subduction database (Fig 17). An improved correlation obtained from full database by [57] demonstrates that upper plate extension usually occurs in subduction zones with higher slab dip angles (Fig 17), consistent with our modeling results (Fig 7).

**Fig 18:**
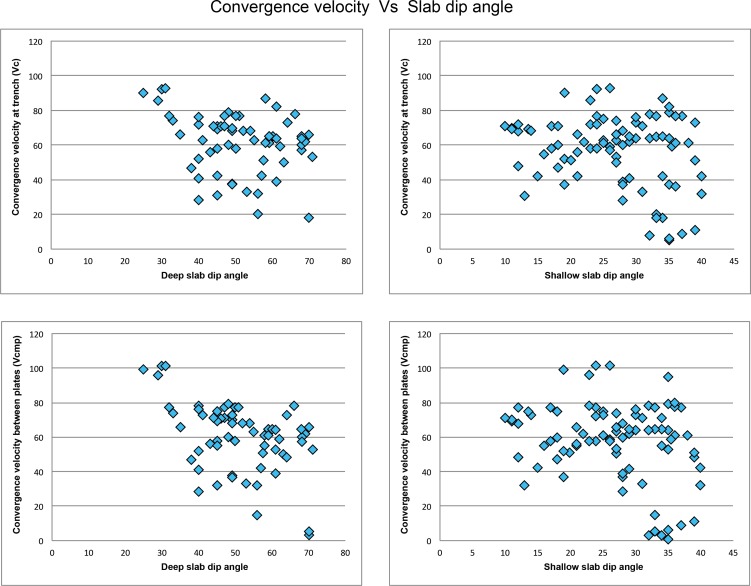
The graphs of convergence velocity with respect to slab dip angle, according to Table 6.

Numerical modeling results show that, greater initial slab dip angle models are generally easier for subduction than the lower initial slab dip angle models. Both the high angle and low angle models get along well with slow subductions. To better understand the relationship between the convergence velocities and slab dip angles, the reduced database is used for analysis (Table 6; Fig 18). For the current subduction systems, the convergence velocity tends to decrease with the deep slab dip angle, whereas no apparent correlation can be found between the shallow slab dip angle and convergence velocity (Fig 18). Model with deep slab dip angle is mainly controlled by the mantle upwelling, whereas complicated stress and strain regime could govern the model of shallow slab dip angle at the accretionary wedge.

The relationship between the convergence velocity and the deep slab angle implies that: (1) The subducting slabs don’t get stuck in slow convergent zones because they have more time to sink into the mantle, trigger mantle upwelling, and produce steeper slab dip angle; (2) Coupling usually occurs in fast subduction zones with low slab dip angle because the compressional stress and strain is enhanced by the fast convergence.

### 4.3 Implications on the subduction zone of the Cathaysia Block

#### 4.3.1 Origin of extensive magmatism in the Cathaysia Block

**Fig 19:**
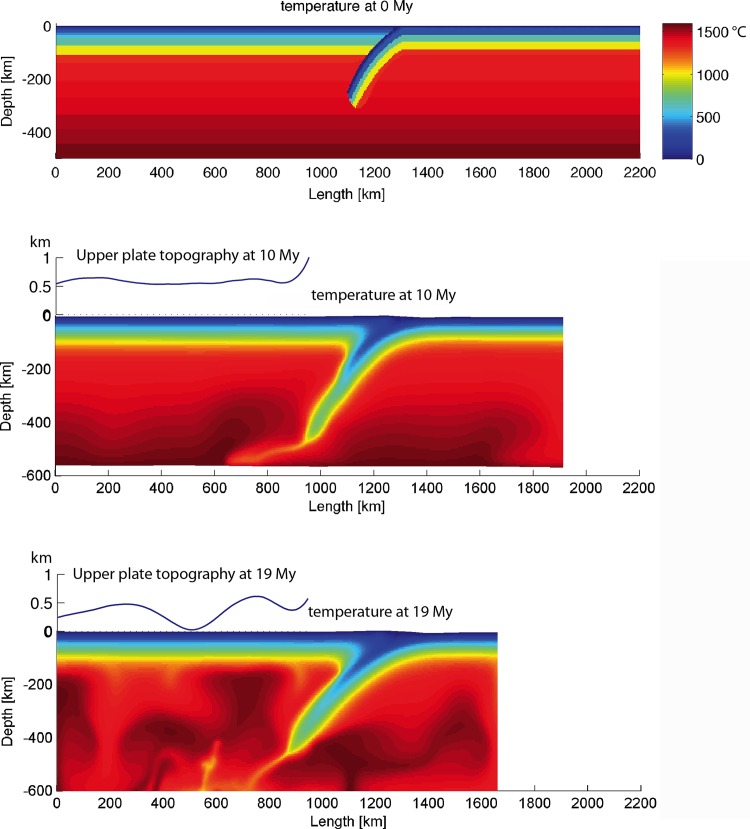
Thermal profiles and the corresponding topography in the areas of interest (upper continental plate) of the reference model at 0 My, 10 My and 19 My.

Widespread Mesozoic granitoids and volcanic rocks in the Cathaysia Block have been considered to result from multi-stage magmatism under low-angle, west-dipping and prolonged subduction of the paleo-Pacific Plate, as revealed by their decreasing age in a sea-ward direction [21]. Chen et al. [68] addressed the origin of Jurassic magmatism as a product of post-orogenic extension and basalt underplating. Zhou et al. [19] proposed that the steepening slab angle is responsible for the large-scale magmatism. These views are consistent with the results of numerical models produced in this study (e.g. “subd_angle2_v1”; Fig 16), in which mantle upwelling is associated with a slab retreat. The models with feasible parameter values for subduction process (Fig 15) were examined for the occurrence of mantle upwelling. It seems that the slab roll-back is relevant to mantle upwelling during the subduction process (Figs 15 and 19). The crustal extension of the Cathaysia Block was probably produced by a slab roll-back coeval to the late stage of the Mesozoic magmatism. As demonstrated in thermal profiles of models with extension in the upper plate (Figs 4 and 19), mantle upwelling under the Cathaysia Block are closely related to the initiation of the extensional regime during the late stage of magmatism. Higher topography is observed above the area of mantle upwelling in modeling results (profile at 19 Ma in Fig 19). The relationship between magmatism and subduction zones has long been extensively discussed [69–72]. Although magmatism is commonly generated during subduction, the formation of volcanic rocks can also postdate active subduction and occur synchronously with uplift, extension or strike-slip motion [73].

In a model comparable to the Cathaysia Block (Fig 19), at the very beginning of the subduction process, the subduction zone is characterized by compressional stress regime from outside to inside, as revealed by the stress distribution in the stress field of the model at 0 My. In the stress field profile at 10 My, the outlined area is mixed with horizontal, oblique and vertical principle stresses, while in the one of 19 My, the outlined area is dominated by vertical principle stresses. The change of the stress distribution shows that the outlined area has undergone a transition from compression to extension, especially in the crustal level.

#### 4.3.2 Subduction velocity in the Cathaysia Block during Mesozoic-Cenozoic

There are some controversies on the convergence velocity in the South China–Paleo-Pacific subduction zone: some publications considered a fast subduction of the paleo-Pacific Plate at a speed between 12 cm/year and 14 cm/year during Late Cretaceous [23,74]; whereas the others estimated the spreading rates of the Pacific Plate ridges as between 4–5 cm/year during Late Cretaceous to Early Paleogene [75–77]. The statistics on the modern subduction zone data showed that the maximum convergence velocity of current subduction zones is at around 10 cm/year (Table 6). This is consistent with our numerical modeling results under the designated setting: it is barely possible to have a convergence velocity as high as 12 cm/year (e.g. model “subd_therm2_v3”; Fig 13).

#### 4.3.3 Compression-extension transition in the continental crust of the Cathaysia Block

**Fig 20:**
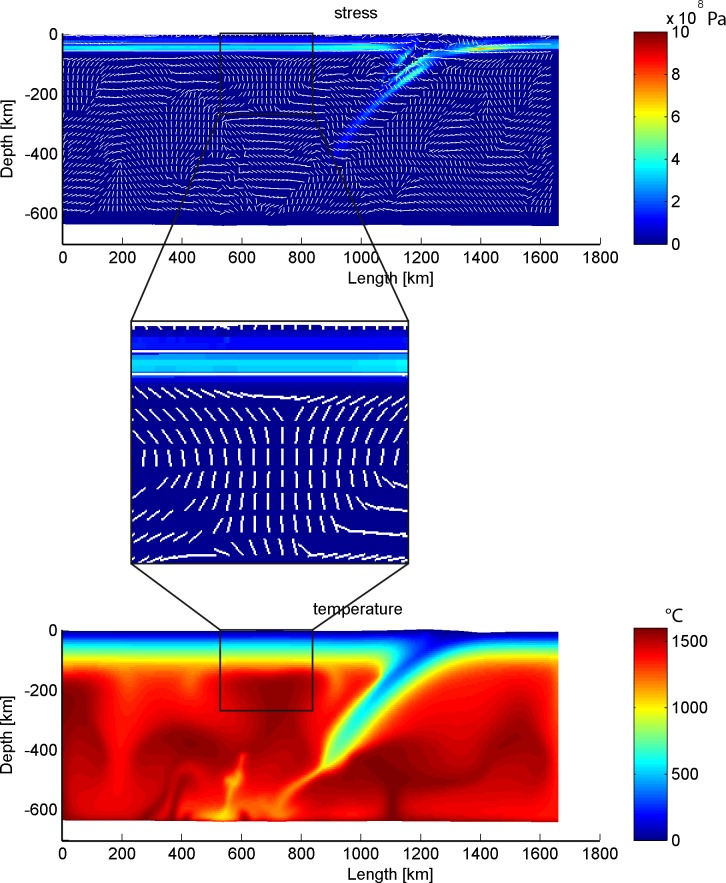
Zooming of stress field in continental crust and the corresponding thermal profile of the reference model at 19 Ma. The profiles of second invariant of deviatoric stress indicate the effective shear stress.

Almost all the feasible subduction models display a phase of slab roll-back, usually accompanied with mantle upwelling. Examination of the stress field of the continental plate in the reference model reveals that horizontal compression dominates during the early stage of the subduction, but it reverts to a horizontal extension in the back-arc region later (Figs 4 and 20). In the reference model, the distribution of sub-vertically directed maximum principal stress in the continental plate in the back-arc region demonstrates that the crust of this region has been in an extensional setting from 19 My, associated with the roll-back of subduction slab and high temperature of the mantle of the back-arc region.

From late Mesozoic, a lot of red bed basins were formed in the Cathaysia Block [78]. It appears that the red-bed basins could have formed during the late stage of the subduction process. At the same time, extensive granitic magmatism, particularly rift-related volcanism, has been generated in the block, which has been thought to result from the mantle upwelling that was triggered by slab roll-back [19]. This may account for the observations of concurrent volcanic rocks in some sedimentary basins in the Cathaysia Block [28,79]. The crustal extension during the late stage of the subduction is also supported by the geological inferences from the apatite fission-track data [80], which shows that the extensional events in the Cathaysia Block started as early as the Late Cretaceous. The change of the stress regime observed in the numerical models is consistent with the extensional events in the continental crust of the Cathaysia Block during the convergent subduction of the paleo-Pacific Plate underneath [17–19,21,24].

## 5. Conclusion

**Fig 21:**
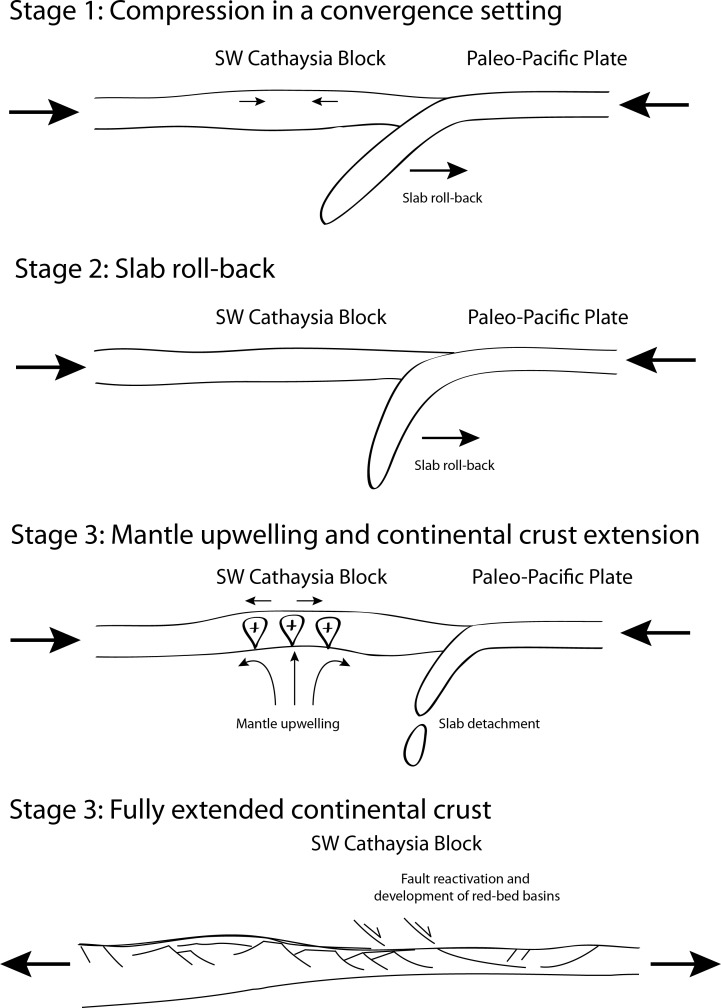
Evolutionary diagram of the Cathaysia Block during the compression-extension transition.

Based on our systematic numerical modeling study of the subduction system, by varying the convergence velocity in of the subduction, the initial slab dip angle, the geothermal gradients of continental and oceanic lithosphere, several major conclusions can be drawn as follows:

1. Parameters, such as low or medium convergence velocity, medium thermal gradients together with various slab dip angles, high thermal gradient for low slab dip angle, and low thermal gradient for high slab dip angle, were critical to an efficient subduction process.
2. Mantle upwelling caused by the diving of slab is believed to make a major contribution to the initiation of continental extension. The utmost amount of mantle upwelling was achieved by rolling-back of the subducting slab at high slab dip angle, which can lead to the compression-extension transition in the continental plate.
3. The extensional regime in the Cathaysia Block can be triggered by the slab roll-back. The slab roll-back can trigger more mantle upwelling and result in extensive magmatism (Fig 21). Such crustal extension could have accounted for concurrent volcanic rocks in red-bed basins and crustal exhumation shortly after the cessation for magmatic episodes in the Cathaysia Block.
